# MM-UNet: A multimodality brain tumor segmentation network in MRI images

**DOI:** 10.3389/fonc.2022.950706

**Published:** 2022-08-18

**Authors:** Liang Zhao, Jiajun Ma, Yu Shao, Chaoran Jia, Jingyuan Zhao, Hong Yuan

**Affiliations:** ^1^ School of Software Technology, Dalian University of Technology, Dalian, China; ^2^ Stem Cell Clinical Research Center, The First Affiliated Hospital of Dalian Medical University, Dalian, China; ^3^ The Affiliated Central Hospital, Dalian University of Technology, Dalian, China

**Keywords:** brain tumor (or Brat), medical image segmentation, multimodality fusion, hybrid attention mechanism, dilated convolution

## Abstract

The global annual incidence of brain tumors is approximately seven out of 100,000, accounting for 2% of all tumors. The mortality rate ranks first among children under 12 and 10th among adults. Therefore, the localization and segmentation of brain tumor images constitute an active field of medical research. The traditional manual segmentation method is time-consuming, laborious, and subjective. In addition, the information provided by a single-image modality is often limited and cannot meet the needs of clinical application. Therefore, in this study, we developed a multimodality feature fusion network, MM-UNet, for brain tumor segmentation by adopting a multi-encoder and single-decoder structure. In the proposed network, each encoder independently extracts low-level features from the corresponding imaging modality, and the hybrid attention block strengthens the features. After fusion with the high-level semantic of the decoder path through skip connection, the decoder restores the pixel-level segmentation results. We evaluated the performance of the proposed model on the BraTS 2020 dataset. MM-UNet achieved the mean Dice score of 79.2% and mean Hausdorff distance of 8.466, which is a consistent performance improvement over the U-Net, Attention U-Net, and ResUNet baseline models and demonstrates the effectiveness of the proposed model.

## Introduction

The brain tumor is the general term for the malignant proliferation of abnormal cells in the brain (1). The continuous expansion of brain tumors compresses the brain nerves, resulting in severe consequences such as headache, nausea, absentmindedness, and even death (2). To treat such diseases, the location of brain tumors must be accurately located, which is impossible without the use of medical image analysis. Manually marking medical images is a cumbersome and error-prone task; thus, establishing an accurate and reliable segmentation method to improve the efficiency of clinical diagnosis and support decision-making is imperative (3, 4). With the continuous development of the deep learning (DL) theory and algorithms in recent years, the data processing ability and generalization ability of neural networks have improved immensely (5, 6). Combining the DL theory with the specific task of medical image analysis, the method of realizing medical image semantic segmentation has been extensively studied (7). Owing to the unique advantages offered by convolutional neural networks (CNNs) in image processing, the U-Net (8) series, Attention-UNet (9), and ResUNet (10) have exhibited excellent results in certain medical image segmentation tasks and have a bright application prospect.

Because the structure of human organs does not vary considerably among individuals and the semantics are straightforward, the low-level features that refer to detailed information in the image and serve as the basis for segmentation are indispensable for medical segmentation. In addition, medical images have the characteristics of complex gradients. Moreover, it is vital to obtain the high-level semantics built on low-level features and use them for the recognition and detection of target or object shapes in images with richer semantic information. They serve as the basis for accurate segmentation and positioning. The successful brain tumor segmentation models developed in recent years are all single-modality models based on deep neural networks (7). However, in practical clinical applications, we often encounter scenarios that require multimodality medical images to assist doctors in making decisions jointly. Imaging examination methods widely used in clinical practice include computed tomography (CT), magnetic resonance imaging (MRI), and positron emission tomography (PET) (11). They employ different imaging principles to obtain the biological structure and metabolic indexes of human organs, tissues, and cells and provide patient information to the attending doctors from diverse perspectives to improve the accuracy of clinical diagnosis. Single-modality networks pose the limitation that they cannot learn the complementary imaging modality characteristics and cross-modal interdependence (12). To overcome this limitation, certain multimodality medical segmentation networks have been developed (11, 13). However, some problems are encountered; for example, the edge description is not sufficiently fine and is insensitive to changes in the target size. Although some modules have been proposed and applied to feature extraction from feature maps, such as Deeplab V3+ (14), they do not take into account that the image becomes very small after multiple rounds of downsampling and that the location and size of the image need to be improved.

**Figure 1:**
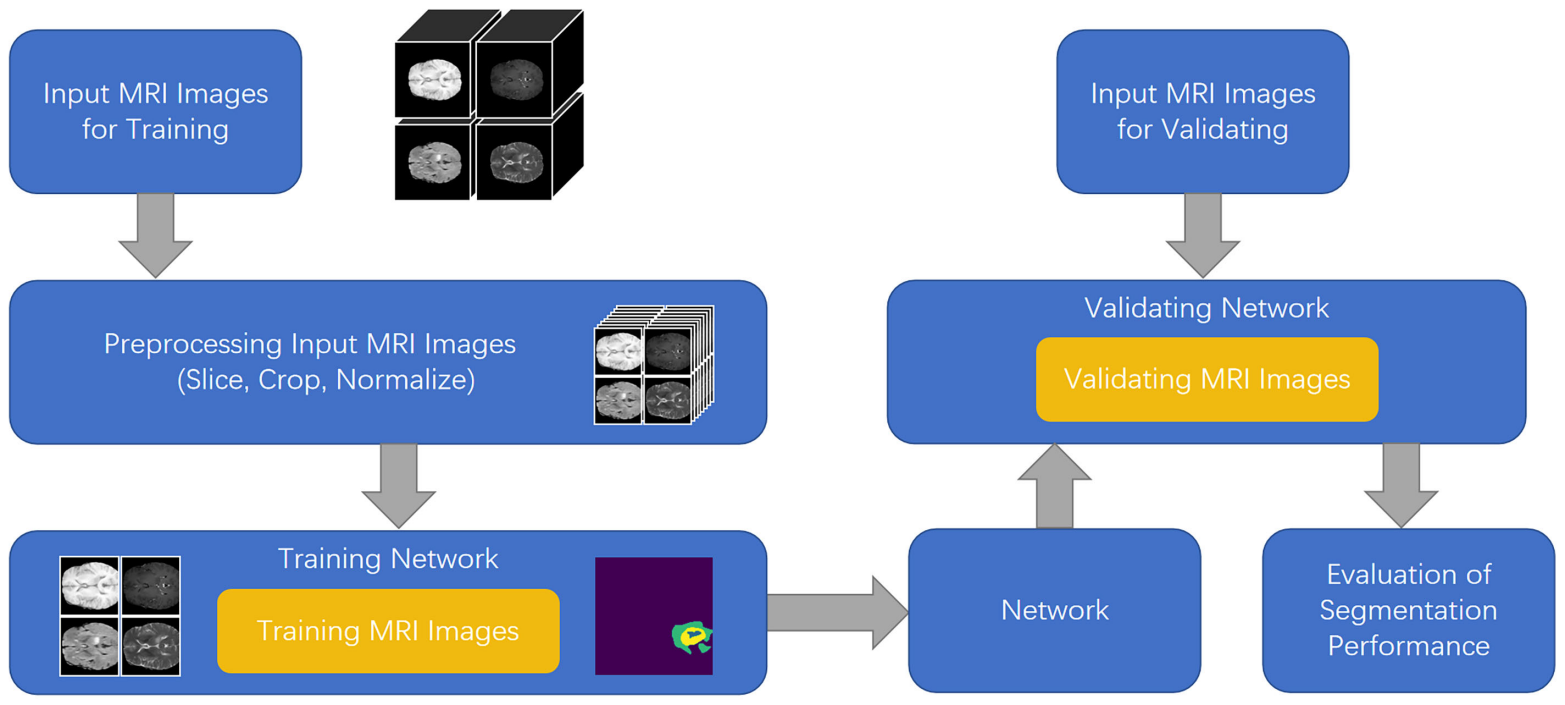
Illustration of brain tumor segmentation pipeline for MM-UNet.

To solve the aforementioned problems, in this study, we developed a feature fusion network based on the multi-encoder and single-decoder structure, named MM-UNet, which extracts the corresponding features from multiple imaging modalities of medical images. The brain tumor segmentation pipeline for MM-UNet is shown in **Figure 1**. In addition, we developed a channel–space hybrid attention block (HAB) to filter the extracted features and remove the redundant information to further improve the network’s ability to utilize the multimodality information comprehensively. Moreover, the encoder often adopts the pooled downsampling method for feature extraction, which may lead to the dilution of the edge details of the segmented region; its negative impact on semantic segmentation cannot be ignored (15). To compensate for the information loss caused by pooled sampling, we introduced dilated convolution (16) with various rates, combined diverse receptive fields without changing the size of the feature map, and depicted the target area at multiple scales to accurately capture the boundary of organs and improve the segmentation accuracy of the model (17, 18).

In summary, the main contributions of the papers are listed as follows: (I) We developed a multi-encoder and single-decoder brain tumor segmentation network called MM-UNet for brain tumor image segmentation. (II) We devised a feature fusion block based on hybrid attention to capture imaging modality features from the two dimensions of channel and space to better focus on the key information in the feature map. (III) We developed a dilated pooling module to capture multiscale information and refine the segmented edge. (IV) We validated the effectiveness of the proposed model on the BraTS 2020 dataset (19), demonstrating improvements over many baseline models.

## Related work

In medical image segmentation, medical images are labeled at the pixel level. However, the various sizes and shapes of human tissues and organs and the unique imaging methods of medical images pose challenges (20, 21). Many methods based on DL have shown promising adaptability (22). The fully CNN (FCN) (23) was one of the earliest attempts to use CNNs for image segmentation. Because the network cancels the fully connected layer, the size of the input image is no longer limited; this considerably widens the adaptation range of the model. The single–encoder-decoder structure in the classical U-Net model adopts the skip connection, which combines the low-level features and high-level semantic information of the same scale feature map (8). Obtaining a large number of medical data is difficult. However, the U-Net model does not require a large amount of data; thus, it has been widely used in medical image segmentation (24).

Although single-modality networks yield satisfactory results, the amount of information available in single-modality images is limited, which cannot comprehensively and systematically reflect the physiological structure of patients (25). To meet the requirements of clinical applications, multimodality segmentation networks have been designed (26). Dolz et al. (27) proposed a multimodality model for the positioning and segmentation of the intervertebral disc, wherein each MRI modality is processed by different paths to make full use of the unique information of each model; in addition, a close connection is adopted between each path so that the model can learn the connection between different imaging modalities independently. Cai et al. (28) proposed a multimodality model for the fusion of MR and CT images that can fuse the features of different imaging modalities without supervision. Xue et al. (29) proposed a method for realizing the interaction between two modal information generated in multimodality CT (PET-CT). They used different encoders for the two modal information and eliminated the misleading features by sharing the downsampling block between the two encoders to realize feature interaction between multiple imaging modalities. Chartsias et al. (30) proposed a multi-input–multi-output FCNN model for MRI synthesis. They fully extracted unique information from each input imaging modality by using a single shared decoder and loss function for each imaging modality. Aygün et al. (31) proposed a multimodality CNN method for brain tumor segmentation by adopting a separate learning method and combination for each imaging modality to improve the network performance. Fang et al. (32) proposed a multimodality brain tumor segmentation framework and mixed fusion of specific imaging modality features. They proposed a new feature fusion scheme that supports the input of a different number of imaging modalities and captures the relevant information of each imaging modality feature through the attention mechanism. For a multimodality medical segmentation network, an appropriate feature fusion strategy must be established (25, 33). The model proposed in the current study adopts a layer-level fusion strategy because it can combine low-level features and high-level semantics without introducing too many parameters, thereby preventing an increase in the complexity of the model.

In addition to selecting the location of feature fusion, determining the complex relationship between modalities to help the segmentation network learn more valuable information is the focus of many medical segmentation models. He et al. (34) proposed ST-Net, which superimposes consecutive video frames into a super-image, performs two-dimensional (2D) convolution on the super-image to obtain local spatiotemporal relationships, and then applies temporal convolution to local spatiotemporal feature maps to establish a global spatiotemporal relationships model. They added a spatial transformer module to preserve the spatially critical information. Oktay et al. (9) added a soft attention mechanism module to the U-Net model. By reweighting and aggregating the information, they selectively ignored part of the information to avoid the interference of irrelevant content. Sinha et al. (35) used the attention mechanism for feature maps of different sizes and proposed the position attention module to capture long-distance dependence so that the model can learn more extensive and rich context information. Hu et al. (36) proposed SE-Net; they added a squeeze-and-excitation (SE) block structure to the network to adaptively extract channel features through the interdependence between channels. Experimental results revealed that, when the SE block is combined with ResNet, the network can learn feature weights according to loss and yields the best results on multiple datasets. Chen et al. (37) used the SE block for medical image segmentation and used this structure in skip connections to learn the channel weights to adaptively learn the contribution of different feature maps to the final segmentation result. They found that this attention structure is effective for medical segmentation tasks. The soft attention mechanism performs reweighted aggregation calculation on the remaining information by selectively ignoring part of the information (38); this characteristic is crucial in multimodality image segmentation tasks. Therefore, in the current study, we proposed a soft attention mechanism module for multimodality image segmentation.

In the medical image segmentation task, accurately locating lesions of various sizes and with irregular edges and contours is challenging (39). The commonly used segmentation algorithms generally use pooled downsampling followed by upsampling to restore the image size so that each pixel can obtain a sizeable receptive field to locate. This process inevitably leads to the loss of image details. Thus, dilated convolution is introduced to expand the receptive field while keeping the spatial resolution unchanged to alleviate the loss of image details caused by the downsampling operation. Yu et al. (40) proposed a network that combines dilated convolution and residual structure to alleviate the problem of spatial feature loss caused by reduced resolution. They demonstrated that networks with dilated structures outperform residual networks without dilated convolutions. Inspired by this, Moreno Lopez et al. (41) employed this dilated residual network for brain tumor segmentation to solve the problem of downsampling loss of resolution and provide efficient multiscale analysis for prediction tasks. They further demonstrated the effectiveness of this structure for brain tumor segmentation through ablation experiments. Chen et al. (42) proposed the ASPP (Atrous Spatial Pyramid Pooling) structure by using the spatial pyramid pooling operation in the target detection model for reference, set dilated rates to capture the multiscale information in the feature map in parallel, and employed global pooling to retain the image-level features. Yang et al. (43) used this ASPP structure for instance-level human analysis to adapt to the problem of large differences in the proportions of different parts of the human body and to better learn the connections between different parts. In addition, inspired by the structure of ASPP, Ni et al. (44) added a SE pyramid pooling block between the encoder and the decoder and increased the size of the receptive field and the ability of multiscale feature fusion; this approach yielded satisfactory performance on several medical segmentation datasets. We imitated this structure and introduced similar modules into the model as an intermediary to connect the two downsampling processes.

## Method

**Figure 2:**
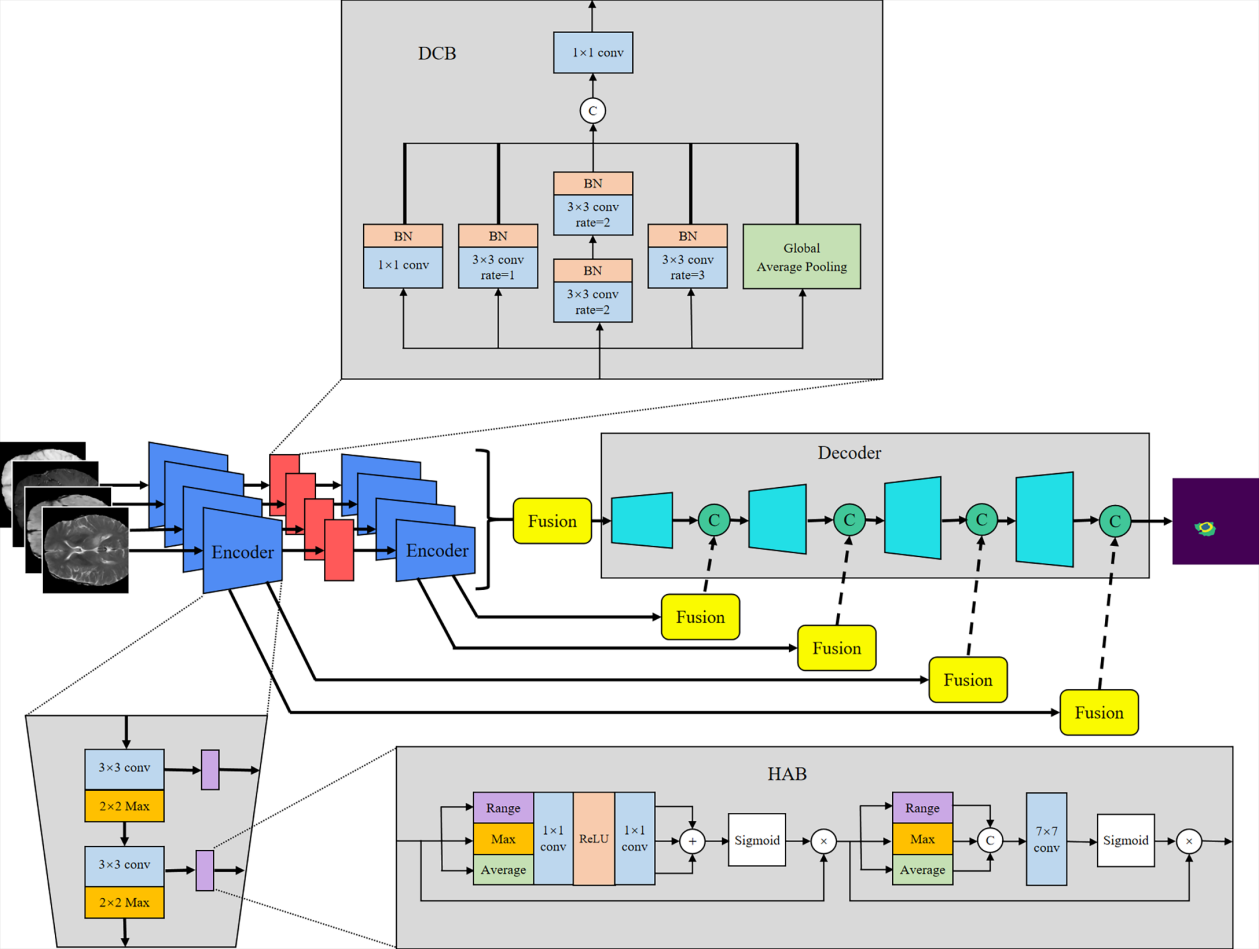
Proposed MM-UNet network architecture.

U-Net (8) offers many advantages in medical image segmentation. Inspired by the structure of U-Net, we developed a multi-encoder and single-decoder model. The network structure is shown in **Figure 2**. Each independent encoder path extracts features from the image of the corresponding imaging modality. Before downsampling, the feature map is transmitted to the channel–space HAB, which suppresses the features with a small amount of information to highlight the image focus and further integrates with the decoder path’s high-level semantics through skip connection. After two rounds of downsampling using the encoder, the feature map is transmitted to the dilated convolution block (DCB) to combine multiscale receptive fields for characterizing brain tumors of various sizes. The decoder gradually restores the high-level semantics after four rounds of downsampling and fusion by the encoder to the pixel-level segmentation results.

### Encoder and decoder

The encoder is used to extract the features of each input imaging modality and obtain independent potential features. The model has four inputs; each encoder takes the image of its corresponding imaging modality as the input and performs continuous downsampling after residual convolution (45). In the residual convolution, two successive 3×3 convolutions are performed to learn the residual, and 1×1 convolution is performed to keep the number of original input channels the same as the residual for element-level addition. The ReLU operation is performed after the 3×3 convolution operation in the residual convolution. The results are transmitted to the corresponding hybrid attention module and the lower sampling layer. Each round of downsampling changes the picture size to half of the original. We used 2×2 max pooling to achieve downsampling. After the above process is repeated four times in the encoder, the characteristic diagram with a total downsampling rate of 16× is outputted. Corresponding to the encoder structure, the decoder also has a four-layer structure. The features of each layer of the encoder are inputted to the hybrid attention module before downsampling, fused with the mask corresponding to other imaging modalities, and then transmitted to the decoder through skip connection to fuse with the features after upsampling again. We adopted channel-level concatenation as the imaging modality fusion method. After fusion, the number of characteristic channels becomes four times that of the original, and the size remains unchanged. The residual convolution input in the decoder is obtained by the channel-level concatenation of the fusion feature of skip connection with the feature of the previous layer. The residual results are added at the element level with the results of the previous layer; then, upsampling is performed to transfer to the next layer. The 2×2 deconvolution implementation used for upsampling changes the picture size to twice the original size. Therefore, after four rounds of downsampling and four rounds of upsampling, the final output picture size is consistent with the original picture. At the end of the network, the number of picture channels is changed into the number of classification categories through one 3×3 convolution. We show the settings of layers in each encoding and decoding stage of the MM-UNet in **Table 1**.

**Table 1:** Details of operations performed, and settings of layers in each encoding and decoding stage of the proposed network.

# Stages	Encoder path	# Output features and Feature size	Decoder path	# Output features and Feature size
1	Input	4@160*160*1	Conv2D [output Layer] [1*1]	160*160*4
	Conv2D [3*3, BatchNorm, ReLU] Conv2D [3*3,BatchNorm, ReLU]	4@160*160*32	Conv2D [3*3, BatchNorm, ReLU] Conv2D [3*3, BatchNorm, ReLU]	160*160*128
	Max Pooling [2*2]	4@80*80*32	Upsampling (Deconvolution layer) [2*2, strides = 2*2]	160*160*256
2	Conv2D [3*3, BatchNorm, ReLU] Conv2D [3*3, BatchNorm, ReLU]	4@80*80*64	Conv2D [3*3, BatchNorm, ReLU] Conv2D [3*3, BatchNorm, ReLU]	80*80*256
	Max Pooling [2*2]	4@40*40*64	Upsampling (Deconvolution layer) [2*2, strides = 2*2]	80*80*512
3	Conv2D [3*3, BatchNorm, ReLU] Conv2D [3*3, BatchNorm, ReLU]	4@40*40*128	Conv2D [3*3, BatchNorm, ReLU] Conv2D [3*3, BatchNorm, ReLU]	40*40*512
	Max Pooling [2*2]	4@20*20*128	Upsampling (Deconvolution layer) [2*2, strides = 2*2]	40*40*1,024
4	Conv2D [3*3, BatchNorm, ReLU] Conv2D [3*3, BatchNorm, ReLU]	4@20*20*256	Conv2D [3*3, BatchNorm, ReLU] Conv2D [3*3, BatchNorm, ReLU]	20*20*1,024
	Max Pooling [2*2]	4@10*10*256	Upsampling (Deconvolution layer) [2*2, strides = 2*2]	20*20*2,048
5	Multimodal Fusion	10*10*1,024	Conv2D [3*3, BatchNorm, ReLU] Conv2D [3*3, BatchNorm, ReLU]	10*10*2,048

### Hybrid attention block

The HAB successively obtains the mask along the channel and spatial dimensions and then multiplies it with the input feature map for adaptive feature optimization. As shown in **Figure 2**, for the feature map *f*∈*ℝ* ^*C*×*H*×*W*^ with the number of input channels C, height H, and width W, we used global average pooling (GAP), global max pooling (GMP), and global range pooling (GRP) calculated by maximum pooling minus minimum pooling to obtain the average value, maximum value, and range of the feature map’s transverse and longitudinal sections, respectively, which reflect the features of the input image in the channel and spatial dimensions. As shown in Equations (1) and (2), in the channel attention part, the feature map obtained using three types of pooling operations is transmitted to the multilayer perceptron layer with shared parameters, and then, the element addition and sigmoid normalization are performed to generate the channel mask. In the spatial attention part, the feature map obtained using three types of pooling operations is used to perform a channel-level concatenation operation, followed by a 7×7 convolution and sigmoid normalization to produce the spatial mask, as shown in Equation (3). Finally, the feature map is multiplied with the two masks successively to obtain the enhanced version, as shown in Equation (4). *F* in the equation represents the feature map of the intermediate process.


(1) $$MLP\left(F\right)=\phi_{2}\left(ReLU\left(\phi_{1}\left(F\right)\right)\right)$$

(2) $$M_{c}\left(F\right)=\sigma \left(MLP\left(GAP_{c}\left(F\right)\right)+MLP(GMP_{c}\left(F\right)+MLP\left(GRP_{c}\left(F\right)\right)\right)$$

(3) $$M_{s}\left(F\right)=\sigma \left(\phi_{3}\left(\left[GAP_{s}\left(F\right);GMP_{s}\left(F\right);GRP_{s}\left(F\right)\right]\right)\right)$$

(4) $$F_{HAB}\left(f\right)=\left(f*M_{c}\left(f\right)\right)*M_{s}\left(f*M_{c}\left(f\right)\right)$$

### Dilated convolution block

The DCB uses multiple dilated rate convolution cores for feature extraction. We referred to the concept of inception structure (46), superimposed small convolution kernels to simulate the receptive field coverage of large convolution cores, and added GAP to retain the global features of the feature map. The structure of the module is illustrated in **Figure 2**.


(5) $$F_{DCB}\left(f\right)=\phi_{5}\left(\left[\phi_{1}\left(f\right);\phi_{2}\left(f\right);\phi_{3}\left(f\right);\phi_{4}\left(f\right);GAP\left(f\right)\right]\right)$$

The process of DCB can be expressed by Equation (5), where *ϕ*_1_ represents 1×1 convolution, *ϕ*_2_ and *ϕ*_3_ represent 3×3 convolutions with dilated rates of 1 and 3, respectively, and *ϕ*_4_ represents 3×3 convolution with a dilated rate of 2 superimposed twice continuously. To ensure that the number of output channels is the same as the number of input channels, a convolution operation *ϕ*_5_ of size 1×1 is performed at the end.

## Experimental setup and result analysis

### Data preparation

We conducted experiments on the BraTS 2020 dataset (47) to verify the effectiveness of the proposed model. The dataset contains 369 patients in the training set and 125 patients in the validation set. It includes multimodal scans of patients with high-grade gliomas and low-grade gliomas. The data for each patient include four MRI image modalities: T1, T1c, T2, and flair (19). The labeled tumors are divided into three nested regions: (a) enhanced tumor region (ET), (b) region composed of enhanced tumor and necrosis (TC), and (c) complete region composed of all tumor tissues (WT). In the experiment, we cropped the 3D image size from 250×240×155 to 160×160×128 and select edits 2D slice, 160×160×1 image as the input to remove some unnecessary background information to pay better attention to the core area of the image.

### Implementation details

The proposed network was implemented in PyTorch with an Nvidia RTX 3090. Considering the resources occupied by the network training process and the running speed, we set the batch size to 8 and trained 15 epochs by using the Adam optimizer (48) with a learning rate of 0.00001. The optimizer and learning rate can ensure that the network converges in a short time to achieve the training purpose. The computational requirements of the proposed model are presented in **Table 2**. Because we aimed to realize the multi-classification segmentation task of sample imbalance, we used a combination of Dice loss (49) and focal loss (50) with the weight of 0.1 and 0.9, respectively, as the loss function. For each classification category, we calculated the loss value of each image.


(6) $$L_{Dice}=1-\frac{2\sum_{i}^{N}y_{i}{y^{\prime }}_{i}+\epsilon }{\sum_{i}^{N}y_{i}+\sum_{i}^{N}{y^{\prime }}_{i}+\epsilon }$$

(7) $${\begin{array}{ll} L_{Focal}=\sum_{i}^{N} & -y_{i}\left(1-{y^{\prime }}_{i}\right) \end{array}}^{\gamma }log\left({y^{\prime }}_{i}\right)-\left(1-y_{i}\right){y^{\prime }}_{i}\gamma log\left(1-{y^{\prime }}_{i}\right)$$

(8) $$L_{Sea}=\alpha L_{Dice}+\beta L_{Focal}$$

**Table 2:** Computational needs of our proposed method on BraTS 2020 dataset.

Method	Params/M	FLOPs/G
Baseline	110.1	69.8
Baseline + Dilated Convolution Block	110.8	70.9
Baseline + Hybrid Attention Block	110.7	69.8
Our Method	111.4	71.0

The loss functions can be expressed using Equations (6) to (8), where *N* is the number of image pixels, *y_i_* and $y_{\;i}^{'}$ are, respectively; the ground truth and predicted value of the pixel *i*; and γ is a modulating factor in the focal loss, which increases the weight of inaccurately classified samples in focal loss compared to cross-entropy loss. When calculating the Dice loss, a very small constant ϵ is added to prevent the divisor from being 0. The final loss value of this point is the average value of the loss value of each category at this point.

We employed the “Dice score” (DSC) and “Hausdorff distance (95%)” (Hausdorff95) obtained from the MICCAI official website to evaluate the model, as shown in Equations (9) and (10), where TP, FP, and FN represent the number of true-positive, false-positive, and false-negative pixels, respectively. We used the Euclidean distance to calculate d in the Hausdorff distance. Hausdorff100 refers to picking the largest of these distances, and Hausdorff95 refers to picking the distance in the 95th percentile. In addition, according to the scoring rules of BraTS, if there is no enhancing tumor in the ground truth of a case, but there is in the predicted results, then the Dice score is 0; otherwise, the score is 1. Therefore, the false positive of enhancing tumors has a great impact on the average score. To avoid the influence of such prediction results on the experimental results, we remove these special values from the obtained data and average the remaining data for calculation.


(9) $$DSC=\frac{2TP}{2TP+FP+FN}$$

(10) $$Hausdorff100=\max\left\{d\left(y,y^{\prime }\right),d\left(y^{\prime },y\right)\right\}$$

### Experiment results

**Figure 3:**
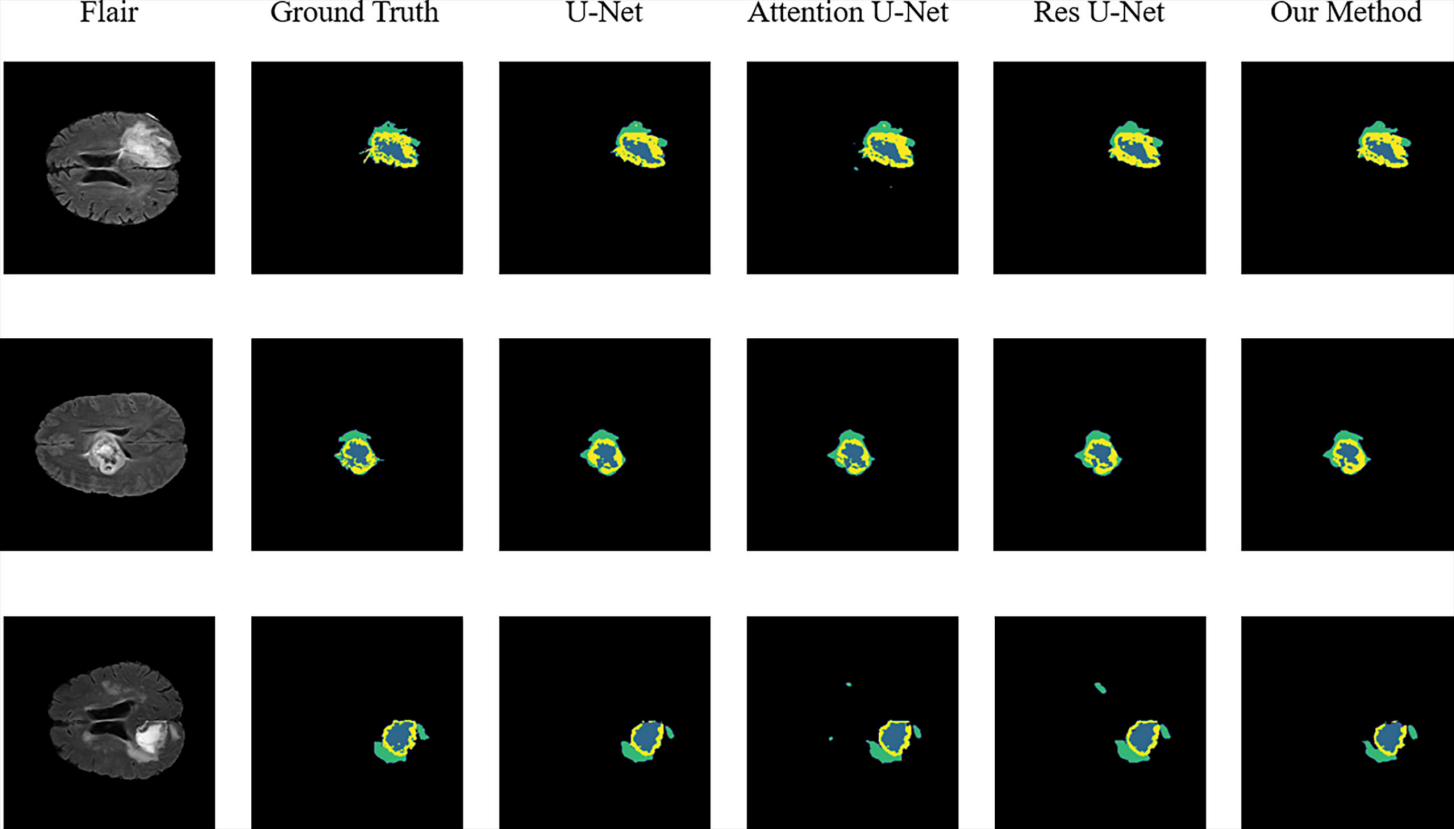
Sample segmentation results. Column 1, input images; column 2, brain tumor ground truth (GT) images; column 3, segmented results of U-Net; column 4, segmented results of Attention U-Net; column 5, segmented results of Res U-Net; column 6, segmented results of our method (ET, blue; TC, yellow + blue; and WT, green + yellow + blue).

To verify the improvement in the segmentation performance of the proposed model, we designed ablation experiments to test the effectiveness of each block and the synergy between them. We regarded the imaging modality without HAB and DCB as the baseline. As can be seen from **Table 3**, the Dice scores of baseline methods in ET, WT, and TC were 71.4%, 84.1%, and 73.7%, respectively, whereas the corresponding Hausdorff distances were 6.554, 12.645, and 8.349, respectively. When the DCB was added to the network, the ET score improved considerably, which shows that the multimodality information improves the depiction of the internal details of the lesion area. Moreover, the WT and TC values decreased slightly. As shown in **Figure 3**, this is due to the small proportion of WT and TC regions in many images in the dataset. Employing only a few rounds of downsampling leads to a great loss of edge information difficulty in restoration. When we introduced the HAB alone, the Dice score increased by 0.6%, and the Hausdorff distance decreased to 1.199 on average, which demonstrates the importance of the multimodality information for the edge contour. Although the performance of the two blocks in different indicators has advantages and disadvantages, when they were used together in the network, most indicators improved in consistency.

**Table 3:** Ablation study of our proposed method on BraTS 2020 dataset.

Method	DSC			Hausdoff95		
	ET	WT	TC	ET	WT	TC
Baseline	0.714	0.841	0.737	6.554	12.645	11.035
Baseline + Dilated Convolution Block	0.765	0.789	0.728	6.586	11.253	15.174
Baseline + Hybrid Attention Block	0.739	0.832	0.738	6.993	8.597	11.046
Our Method	0.762	0.850	0.765	6.389	8.243	10.766

To validate the performance of the proposed model, we compared the segmentation results of the proposed model with that of the original U-Net, Attention U-Net, and ResUNet models because they are commonly employed for medical image segmentation. **Table 4** presents their segmentation results on the BraTS 2020 dataset for comparison. The proposed model exhibited superior performance compared with the three aforementioned models in terms of each specific index. To evaluate the model performance intuitively, we randomly selected some visual segmentation results from the BraTS 2020 dataset. As can be seen in **Figure 3**, the original U-Net model exhibited inferior performance for densely distributed area segmentation. Although Attention U-Net and ResUNet roughly divided the structure of overlapping areas, they failed to accurately capture some prominent edge parts because of the lack of multiscale information obtained by the network. The proposed model yielded more accurate results for the location of the lesion area, and the edge contour was more explicit. Moreover, its segmentation result was remarkably close to the manually marked ground truth. The control experiment results on DCB and loss function are presented in **Table 5**.

**Table 4:** Comparative results on BraTS 2020 dataset.

Method	DSC			Hausdoff95		
	ET	WT	TC	ET	WT	TC
U-Net	0.707	0.825	0.732	9.035	12.174	14.361
Attention U-Net	0.710	0.809	0.703	13.983	22.887	22.799
ResUNet	0.723	0.813	0.738	6.613	9.075	11.225
Our Method	0.762	0.850	0.765	6.389	8.243	10.766

**Table 5:** Control experiment on BraTS 2020 dataset.

Method	DSC			Hausdoff95		
	ET	WT	TC	ET	WT	TC
Spatial first	0.737	0.802	0.713	41.072	7.990	30.989
Channel and spatial parallelism	0.737	0.851	0.768	8.381	7.846	12.359
DCB after quartic downsampling	0.773	0.854	0.750	6.573	14.199	11.409
Dice loss as loss function	0.745	0.844	0.729	6.001	8.206	11.021

## Discussion

Brain tumor segmentation is an important part of medical image processing, its purpose is to assist doctors to make accurate diagnosis and treatment, and it has important practical value in the field of clinical brain medicine. Segmentation of brain tumor images remains a challenging topic due to the high variability in the size, shape, and location of brain tumors, as well as limited and unbalanced brain tumor image data.

To address the above problems, we designed and implemented a segmentation network based on a multi-encoder and single-decoder structure, named MM-UNet, for multimodal brain tumor image segmentation. We devised a feature fusion block based on hybrid attention to better focus on the key information in the feature map. We developed a dilated pooling module that enables models to focus on more valuable information. To verify whether the proposed model has the ability to address the above problems, we conducted ablation and contrast experiments. Experimental results showed that the network is better than U-Net, Res U-Net, and Attention U-Net that are commonly used in medical segmentation.

Although some solutions to these problems have been proposed in the field of image segmentation, they still have problems. After the image is subjected to successive downsampling operations, the image size is changed and the existing modules are not suitable for small image sizes. We redesigned a feature extraction module and explored the effect of this module on the model performance at different positions. Furthermore, our proposed attention module incorporates more information to provide more choices for the network. Our model may play a role in future clinical diagnosis, as this structure could help physicians better locate tumor regions of different sizes in brain tumor image analysis and to more clearly delineate the boundaries of these tumor regions.

Some problems were encountered during the construction of the model and during the experiments. We performed some control experiments to analyze the factors that may affect the experimental results to determine the optimal solution for the proposed network. For the HAB, the sequence of placing the channel and space parts affected the segmentation accuracy of the model. We refer to the structure of the convolutional block attention module and adopt the placement sequence of the channel followed by space. However, parallel placement of the two parts produced superior performance in the case of some indicators, as can be seen in **Figure 3**; this may be because the image data of specific patients are consistent in the dimensions of channel and space; the two are equally crucial for segmentation precision, and the use of serial sequence leads to interactions between them.

In addition, we experimentally studied the position of the DCB. The DCB is typically placed at the intersection of the encoder and decoder as a bridge between them. The position where we placed the DCB yielded a higher segmentation accuracy, as can be seen in **Figure 3**. This may be because the closer we are to the bottom of the encoder, the smaller the feature map outputted by the convolution block, which is equivalent to expanding the receptive field in a disguised form. The perception of large-scale brain tumor areas was our primary aim; thus, we ignored small areas and details. Selecting the best position may be related to the number of rounds of downsampling using the encoder and the size of the input image. We selected the appropriate position according to the characteristics of the brain tumor area; however, this affects the accuracy of the segmentation in some cases.

As the training target indicator of the model, the loss function has a considerable effect on the change direction of the parameters. The performance of the model may vary for training performed under the guidance of different loss functions. Although the pure Dice loss can match the evaluation index well, it is unfavorable for gradient backpropagation and makes the training unstable. Therefore, we used the combination of Dice loss and focal loss because of their flexibility in dealing with unbalanced sample datasets and smoothing the training curve. We compared the results with that of the model trained using a pure Dice loss function, and the results are shown in **Figure 3**, verifying our conjecture.

## Conclusions

In this study, we developed a multimodality fusion network based on the hybrid attention mechanism for brain tumor segmentation. In the proposed model, the encoders extract the image features of diverse imaging modalities, which are refined by the HAB and fused with the features obtained in the upsampling stage. In addition, the DCB is used to describe the multiscale features to generate more accurate segmentation results. The experimental results revealed that, compared with other models such as U-Net, the proposed model achieves superior results, as can also be seen intuitively from the segmentation graph.

## Data availability statement

Publicly available datasets were analyzed in this study. This data can be found here: https://ipp.cbica.upenn.edu.

## Author contributions

LZ and JM contributed to conception and design of the study. YS organized the database. CJ performed the visualization. JM wrote the first draft of the manuscript. HY and JZ supervised the project. All authors contributed to manuscript revision, read, and approved the submitted version.

## Funding

This work is supported by the National Natural Science Foundation of China (61906030), the Science and Technology Project of Liaoning Province (2021JH2/10300064), the Natural Science Foundation of Liaoning Province (2020-BS-063), and the Youth Science and Technology Star Support Program of Dalian City (2021RQ057).

## Conflict of interest

The authors declare that the research was conducted in the absence of any commercial or financial relationships that could be construed as a potential conflict of interest.

## Publisher’s note

All claims expressed in this article are solely those of the authors and do not necessarily represent those of their affiliated organizations, or those of the publisher, the editors and the reviewers. Any product that may be evaluated in this article, or claim that may be made by its manufacturer, is not guaranteed or endorsed by the publisher.
